# The Need for Trained Workhouse Matrons

**Published:** 1908-01-11

**Authors:** 


### Nursing Administration.

### THE NEED FOR TRAINED WORKHOUSE MATRONS.

Attention has again been drawn in the recent report of the Local Government Board to the difficulty experienced in small workhouses in rural districts with regard to the nursing of the occasional sick persons who are received as inmates. Very often there is no one who can be considered really ill. At most there may be two or three requiring the attentions of a nurse. To keep a nurse constantly engaged in the workhouse on the chance of these cases occurring is costly. But the cost is not the most serious point to be considered. There is little to attract a woman who is interested in her work, and those nurses who are satisfactory seldom stay long. It is by no means easy to engage temporary nurses for such work, and hence a never-ending source of anxiety confronts the Guardians. Mr. James Lowry suggests that the difficulty would be removed if the workhouse matron were herself a trained nurse, and were this suggestion accepted generally in rural unions, we are disposed to think great benefit would ensue, not merely to the sick, but to the bulk of the workhouse population. It might also prove the solution of the outstanding difficulties liable to occur between the matron of the workhouse and the superintendent of the sick wards attached to it. Friction is never so prone to arise as when the field of authority is too small to give scope for more than one person in command. And the appointment of a workhouse matron who was herself a trained nurse and qualified to superintend the whole establishment would work beneficially by placing one official in charge instead of two.

There are other reasons why it is highly desirable to see trained nurses appointed to the post of workhouse matron. The training given in hospitals and in good Poor-law infirmaries is the best possible preparation afforded at the present time to intending matrons, whatever class of institution they may be called on to superintend. The power of carrying out orders intelligently, of working under closely defined regulations, of dealing with subordinates, of keeping order among numbers, of making exact observations, of maintaining rigid cleanliness and order in every department of work, of doing small things as well as they can possibly be done—all these are qualities which the matron of orphanage, workhouse, refuge, or home of any description ought to possess, and nowhere can she learn them so well as in the well-ordered wards of a hospital or infirmary. In addition to this hardly a day passes in any institution in which the matron will not find it a distinct advantage to possess a knowledge of the laws of health, to be able to distinguish trifling ailments from real illness, and deal herself promptly with the one or the other. We have not touched on the aspect of humanity. There can be no doubt that gentler methods are learned in attendance at the bedside of sick and aged than in the sterner training ground afforded to the would-be workhouse matron among the ablebodied. But it would be unfair to the present generation of workhouse matrons who are not trained nurses to impute to them any lack of kindliness to those under their charge. In numerous instances their administration reveals unmistakably their keen sympathy with the poor. In urging that candidates for these posts should be encouraged to undergo training as nurses we desire to render the exceptions to the rule of humanity more rare, and to lend these posts which are often influential to a degree little guessed at by the public a higher standing. There can be no doubt that were nursing qualifications considered in making the appointments, a better choice of candidates would be obtained and many difficulties in the smooth working of these establishments would be obviated.

Truth to tell the choice of matron is too often sacrificed to the obvious convenience of having a married couple as master and matron. When this is insisted on it generally means that the best has to be made of one or other of the pair, and since an indifferent master is a serious danger to the discipline of the establishment, naturally the wife of a good master stands an excellent chance of getting the post of matron. Now if nursing qualifications were a *sine qua non*, such married candidates would take good care to obtain the necessary training, and the extent to which they would personally benefit may be held to equal in value the benefit accruing also to the institution.

We hear a great deal perennially about "pauper helps" in rural workhouses, and no one can be found to say very much in their favour. But it must be considered that in lonely country districts it is absolutely impossible to dispense with the aid which certain workhouse inmates are well able to render in the sick wards. Their number has, in fact, increased of late in more than one part of the country. With a trained matron at the head of the workhouse the evil can be reduced to a minimum. Instead of despatching to the sick wards people for whom she herself has personally no use, she would be able not merely to pick out those who showed an aptitude for helping the nurse, but would speedily impart elementary principles of training to these assistants, so that they were intelligent in the performance of their duties, and begin to take pride in their promotion. Much more could be done in small workhouses in admitting the women to a share in every-day duties if the matron were trained, and as a natural consequence possessed the art of training others.

But would trained nurses apply for the posts we have described? And the women trained in Poor-law infirmaries are a more numerous company year by year. The post of superintendent nurse for the sick wards is exposed to continual vexations arising out of the relations between this functionary and the master and mistress of the workhouse. Were the matron herself the superintendent, an excellent position would be thrown open to capable nurses, and we can have little doubt that the administration of the rural workhouses would undergo a marked amelioration.

